# Criteria for identifying potentially resectable patients with initially oncologically unresectable hepatocellular carcinoma before treatment with lenvatinib plus an anti–PD–1 antibody

**DOI:** 10.3389/fimmu.2022.1016736

**Published:** 2022-11-25

**Authors:** Bin Xu, Xiao-Dong Zhu, Ying-Hao Shen, Jin-Jin Zhu, Jie Liu, Mei-Ling Li, Pei-Wen Tang, Jian Zhou, Jia Fan, Hui-Chuan Sun, Cheng Huang

**Affiliations:** Department of Liver Surgery and Transplantation, Liver Cancer Institute and Zhongshan Hospital, Fudan University, Shanghai, China

**Keywords:** anti-PD-1 antibody, combination drug therapy, conversion therapy, hepatocellular carcinoma, lenvatinib, potentially resectable

## Abstract

**Background:**

Conversion therapy is feasible in patients with oncologically unresectable hepatocellular carcinoma (HCC). However, it is challenging to prospectively identify patients who are more likely to achieve successful conversion before initiating systemic therapy, either alone or combined with locoregional therapy.

**Methods:**

Criteria for identifying potentially resectable patients with initially oncologically unresectable HCC before treatment with lenvatinib plus an anti-PD-1 antibody were proposed based on real-world evidence. Multivariate Firth logistic regression was used to validate the proposed criteria in a retrospective cohort of consecutive patients with advanced HCC, who received combination therapy with lenvatinib plus an anti-PD-1 antibody between September 2018 and September 2021.

**Results:**

The proposed criteria were as follows: (1) Eastern Cooperative Oncology Group performance status of 0 or 1; (2) Child-Pugh class A; (3) intrahepatic tumors confined to one lobe (left, right, or middle lobe), or present in one lobe alongside a single tumor with diameter ≤5 cm or up to three tumors each with diameter ≤3 cm in the remaining lobes, with R0 resection achievable by hemihepatectomy, alone or combined with locoregional therapy to the remaining lobes during surgery; and (4) no portal vein tumor thrombus involving the contralateral liver lobe or reaching the superior mesenteric vein, no hepatic vein tumor thrombus involving more than two major hepatic vein branches on the tumor side, and no tumor thrombus of the inferior vena cava reaching the atrium. Firth logistic regression confirmed the criteria were an independent predictor of surgery following conversion therapy with lenvatinib plus an anti-PD-1 antibody.

**Conclusions:**

This study proposed and validated criteria for identifying patients with initially oncologically unresectable HCC who are potentially resectable when treated with combination therapy with lenvatinib plus an anti-PD-1 antibody. The proposed criteria could help standardize conversion therapy studies in advanced HCC.

## Introduction

Hepatocellular carcinoma (HCC) is one of the most common malignant tumors and one of the leading causes of cancer-related death, both worldwide and in China (1, 2). Most patients with HCC are diagnosed at an advanced stage, when radical surgery is not possible and treatment options are limited (3). HCC can be deemed unresectable for surgical or oncologic reasons (4). Patients with HCC who cannot undergo hepatectomy safely due to poor general condition, inadequate liver function reserve, or insufficient future liver remnant volume (FLRV) are considered surgically unresectable, whereas those in whom surgery is not expected to provide superior outcomes compared with non-surgical treatments are considered oncologically unresectable. However, criteria for defining oncologically unresectable HCC are not so well-established as those for surgically unresectable HCC (5, 6).

Some patients with initially oncologically unresectable HCC can undergo surgery after downstaging (i.e. conversion) therapy. For example, locoregional treatments, such as transarterial chemo- or radioembolization, have been shown to downstage the tumor and thereby enable surgical excision (7, 8). Following remarkable recent progress in targeted therapy and immunotherapy for HCC, which provides improved objective response rates (ORRs), a range of studies have reported that systemic therapy with or without locoregional therapy is a feasible conversion strategy for patients with initially unresectable and advanced HCC (6, 9–15). However, differences in patient selection, criteria for surgical resection, and conversion therapy regimens have led to discordance in reported conversion rates and hamper comparisons between studies. Therefore, in the setting of conversion therapy for initially oncologically unresectable HCC patients, criteria for identifying ‘potentially resectable’ patients, criteria for successful downstaging, and the optimal treatment approach to maximize successful conversion are critical issues on which a consensus should be reached (4).

For patients with oncologically unresectable HCC, successful conversion therapy requires selection of patients with a suitable tumor burden and administration of effective pre-operative anti-tumor therapy. Since ORRs with current systemic therapies for advanced HCC are unlikely to show substantial improvement in the near future, optimizing the identification of patients with suitable tumor burden (i.e. potentially resectable patients) is a key strategy for increasing conversion rates. Meanwhile, patients who are less likely to achieve resectability may benefit from alternative treatment strategies associated with higher ORRs, such as systemic therapy plus locoregional therapy.

Combination therapy with lenvatinib plus pembrolizumab for the first-line treatment in advanced HCC showed a promising ORR of 36% per Response Evaluation Criteria in Solid Tumors (RECIST) v1.1 in the phase Ib KEYNOTE-524 study, with promising progression-free survival (PFS) and overall survival (OS) (16). Similar ORRs have been reported for lenvatinib in combination with a range of different PD-1 antibodies (17). Although LEAP-002 study (lenvatinib plus pembrolizumab vs. lenvatinib as first-line therapy for advanced HCC, NCT03713593) is a negative trial in terms of PFS and OS, it is also noted that ORR was much higher in the combination arm than the lenvatinib monotherapy arm (18), which implies there may be a role of this combination therapy in neoadjuvant therapy. Indeed, preliminary investigation suggested a great value of this combination treatment in either conversion therapy or neoadjuvant therapy settings (11, 14, 19). A multicenter prospective clinical trial has been initiated in China to further investigate the efficacy of this combination in neoadjuvant setting (NCT05389527).

In this study, we used data from a real-world cohort of patients with initially oncologically unresectable HCC, who received combination therapy with lenvatinib plus an anti-programmed death-1 (anti-PD-1) antibody, to propose and validate criteria for identifying patients who are potentially resectable when treated with this regimen.

## Material and methods

### Cohort for criteria validation

Data from consecutive patients with unresectable or advanced HCC, who received combination therapy with lenvatinib plus an anti-PD-1 antibody between September 2018 and September 2021 at Zhongshan Hospital, Fudan University, Shanghai, China, were retrospectively collected.

Patients with China liver cancer (CNLC) stage IIb, IIIa or IIIb disease (20) (i.e. HCC unresectable mainly for oncologic reasons, Barcelona Clinic Liver Cancer stage B or C disease), and at least one tumor response assessment after initiating combination therapy were eligible. Patients with incomplete clinicopathologic data or additional anti-tumor treatment after the initiation of combination therapy were excluded.

All patients received combination therapy with lenvatinib (8 mg/day, orally) plus an anti-PD-1 antibody. The interval between initiation of lenvatinib and an anti-PD-1 antibody was within one week. One of the following anti-PD-1 antibodies was intravenously administered: nivolumab 3 mg/kg (21), or camrelizumab 200 mg (22) every 2 weeks; or pembrolizumab 200 mg (23), sintilimab 200 mg (24), toripalimab 240 mg (25), or tislelizumab 200 mg (26) every 3 weeks. Similar ORRs have been reported for lenvatinib in combination with a range of different anti-PD-1 antibodies (17). Tumor response was assessed every 2 months (± 2 weeks) *via* computed tomography or magnetic resonance imaging according to RECIST v1.1 and modified RECIST (mRECIST), and summarized as best overall response. After treatment, patients were assessed for eligibility for liver resection according to previously published criteria (11). Briefly, patients were classified as having resectable HCC after combination therapy if (1) R0 resection could be achieved with sufficient remnant liver volume and function (2), intrahepatic lesions were evaluated as partial response or stable disease for at least 2 months (3), no severe or persistent adverse effects occurred from systemic therapy, and (4) no contraindications for hepatectomy existed.

The study protocol was approved by the Zhongshan Hospital Research Ethics Committee (Approval Number: B2020-177R). All patients provided written informed consent before initiating combination therapy. The study was conducted in accordance with the principles of the Declaration of Helsinki.

### Follow-up

Patients were followed every 60 days (± 7 days) after initiation of combination therapy. OS was calculated from the date of first dose of drug to death from any cause, or censored on the last follow-up. Event-free survival (EFS) was calculated from the date of first dose of drug to the first documented disease progression, recurrence or death from any cause.

### Statistical analysis

Categorical variables were expressed as counts and percentages, and compared using Pearson’s χ^2^ test, Fisher’s exact test, or the Mann-Whitney U test, as appropriate. Continuous variables were expressed as means (± standard deviations) or medians (interquartile ranges [IQR]) and were compared using Student’s t-test or the Mann-Whitney U test, as appropriate. Firth logistic regression was used to identify independent predictors of surgery (27, 28). Clinicopathologic features with a *P* value of <0.2 in univariate analyses were included in multivariate analyses. Survival curves were calculated using the Kaplane-Meier method and compared using the log-rank test. A *P* value of <0.05 was considered statistically significant. All statistical analyses were performed using R software (version 4.1.2; R Project for Statistical Computing).

## Results

### Patient characteristics

Of 203 patients included, 187 had CNLC stage IIb, IIIa or IIIb disease and were eligible for validation of our proposed criteria. Baseline demographic and disease characteristics are shown in **Table 1**. Twenty-nine patients (15.5%) underwent R0 hepatectomy, indicating a conversion rate which was consistent with our previous study (11).

**Table 1:** Clinicopathologic features and response in patients who did or did not undergo surgery.

Variables	All patients (n = 187)	No surgery (n = 158)	Surgery (n = 29)	*P* value*
Age, years, mean ± standard deviation	55.26 ± 11.4	55.44 ± 11.69	54.28 ± 9.81	0.573
Sex, n (%)				1
Female	23 (12.3)	20 (12.7)	3 (10.3)	
Male	164 (87.7)	138 (87.3)	26 (89.7)	
ECOG PS, n (%)				0.366
0–1	177 (94.7)	148 (93.7)	29 (100)	
2	10 (5.3)	10 (6.3)	0 (0)	
Child-Pugh class, n (%)				0.139
A	171 (91.4)	142 (89.9)	29 (100)	
B	16 (8.6)	16 (10.1)	0 (0.0)	
HBsAg, n (%)				1
Negative	33 (17.6)	28 (17.7)	5 (17.2)	
Positive	154 (82.4)	130 (82.3)	24 (82.8)	
HBV DNA, n (%)				0.932
≤10^3^/mL	87 (46.5)	71 (44.9)	16 (55.2)	
>10^3^/mL	78 (41.7)	65 (41.1)	13 (44.8)	
N/A	22 (11.8)	22 (13.9)	0 (0.0)	
BCLC stage, n (%)				0.161
B	37 (19.8)	28 (17.7)	9 (31.0)	
C	150 (80.2)	130 (82.3)	20 (69.0)	
CNLC stage, n (%)				0.255
IIb	37 (19.8)	28 (17.7)	9 (31.0)	
IIIa	75 (40.1)	65 (41.1)	10 (34.5)	
IIIb	75 (40.1)	65 (41.1)	10 (34.5)	
Extrahepatic disease, n (%)				0.641
No	112 (59.9)	93 (58.9)	19 (65.5)	
Yes	75 (40.1)	65 (41.1)	10 (34.5)	
Macrovascular invasion, n (%)				0.2
No	86 (46.0)	69 (43.7)	17 (58.6)	
Yes	101 (54.0)	89 (56.3)	12 (41.4)	
AFP, ng/mL, median (IQR)	609 (11.65, 15692.5)	942.5 (12.72, 16148.75)	283.4 (8.8, 10748)	0.416
AFP, n (%)				0.683
≤400 ng/mL	87 (46.5)	72 (45.6)	15 (51.7)	
>400 ng/mL	100 (53.5)	86 (54.4)	14 (48.3)	
PIVKA-II, mAU/mL, median (IQR)	3697 (258.5, 25285)	3831 (255.75, 25099.75)	3258 (328, 34657)	0.899
PIVKA-II, n (%)				0.911
≤1000 mAU/mL	66 (35.3)	55 (34.8)	11 (37.9)	
>1000 mAU/mL	121 (64.7)	103 (65.2)	18 (62.1)	
Diameter of intrahepatic tumors, median (IQR)	9.6 (4.94, 14.4)	9.7 (4.56, 14.4)	9.6 (7, 14.3)	0.535
Treatment line, n (%)				0.305
1	153 (81.8)	126 (79.7)	27 (93.1)	
2	32 (17.1)	30 (19)	2 (6.9)	
3	2 (1.1)	2 (1.3)	0 (0)	
Anti-PD-1 antibody used, n (%)				0.627
Camrelizumab	59 (31.6)	47 (29.7)	12 (41.4)	
Nivolumab	11 (5.9)	10 (6.3)	1 (3.4)	
Pembrolizumab	21 (11.2)	16 (10.1)	5 (17.2)	
Sintilimab	68 (36.4)	60 (38.0)	8 (27.6)	
Tislelizumab	13 (7.0)	12 (7.6)	1 (3.4)	
Toripalimab	15 (8.0)	13 (8.2)	2 (6.9)	
BOR per RECIST v1.1, n (%)				<0.001
CR	4 (2.1)	3 (1.9)	1 (3.4)	
PR	45 (24.1)	31 (19.6)	14 (48.3)	
SD	87 (46.5)	73 (46.2)	14 (48.3)	
PD	51 (27.3)	51 (32.3)	0 (0.0)	
Objective response per RECIST v1.1, n (%)				0.002
No	138 (73.8)	124 (78.5)	14 (48.3)	
Yes	49 (26.2)	34 (21.5)	15 (51.7)	
Disease control per RECIST v1.1, n (%)				<0.001
No	51 (27.3)	51 (32.3)	0 (0.0)	
Yes	136 (72.7)	107 (67.7)	29 (100)	
BOR per mRECIST, n (%)				<0.001
CR	9 (4.8)	6 (3.8)	3 (10.3)	
PR	61 (32.6)	41 (25.9)	20 (69)	
SD	66 (35.3)	60 (38)	6 (20.7)	
PD	51 (27.3)	51 (32.3)	0 (0.0)	
Objective response per mRECIST, n (%)				<0.001
No	117 (62.6)	111 (70.3)	6 (20.7)	
Yes	70 (37.4)	47 (29.7)	23 (79.3)	
Disease control per mRECIST, n (%)				<0.001
No	51 (27.3)	51 (32.3)	0 (0.0)	
Yes	136 (72.7)	107 (67.7)	29 (100)	
Change from baseline per RECIST v1.1, median (IQR)	-0.16 (-0.32, 0)	-0.11 (-0.32, 0)	-0.26 (-0.39, -0.17)	0.004
Change from baseline per mRECIST, median (IQR)	-0.3 (-0.64, 0)	-0.18 (-0.49, 0)	-0.65 (-0.82, -0.33)	<0.001
Surgery after therapy, n (%)				–
No	158 (84.5)	–	–	
Yes	29 (15.5)	–	–	

*P values are for the comparisons between patients who underwent surgery vs those who did not.

AFP, a-fetoprotein; BCLC, Barcelona Clinic liver cancer; BOR, best overall response; CNLC, China liver cancer; CR, complete response; ECOG, PS Eastern Cooperative Oncology Group performance status; HBsAg, hepatitis B surface antigen; HBV, hepatitis B virus; IQR, interquartile range; mRECIST, modified Response Evaluation Criteria in Solid Tumors; N/A, not available; PD-1, programmed death-1; PD, progressive disease; PIVKA-II, protein induced by vitamin K absence or antagonist-II; PR, partial response; RECIST, Response Evaluation Criteria in Solid Tumors; SD, stable disease.

### Surgery following combination therapy provided survival benefit

**Figure 1:**
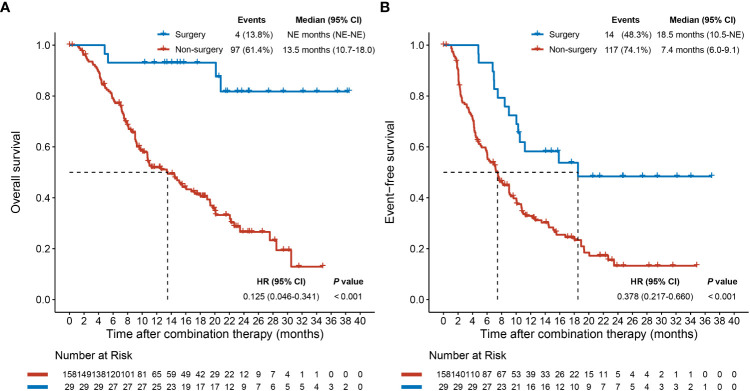
Overall survival **(A)** and event-free survival **(B)** plots after combination therapy initiation for patients who underwent or did not undergo conversion surgery. CI, confidence interval; HR, hazard ratio; NE, not evaluable.

As the data cutoff on April 22, 2022, median follow-up was 11.3 (IQR: 7.1–19.8) months. Patients in the surgery group were associated with a significantly longer median OS or median EFS than those in the non-surgery group (median OS: not evaluable (NE) [95% CI: NE–NE] months vs. 13.5 [95% CI: 10.7–18.0] months, hazard ratio [HR] [95% CI]: 0.125 [0.046–0.341], *P* < 0.001; median EFS: 18.5 [95% CI: 10.5–NE] months vs. 7.4 [95% CI: 6.0–9.1] months, HR [95% CI]: 0.378 [0.217–0.660], *P* < 0.001; **Figure 1**), which indicated that surgery following conversion therapy can provide a survival benefit.

### Routine clinicopathologic features at baseline were not associated with surgery

We first compared clinicopathologic features before combination therapy and tumor responses after combination therapy between patients who did or did not undergo surgery (**Table 1**). Individual routine clinicopathologic features at baseline were not correlated with surgery and could not therefore be used to identify potentially resectable patients who were initially oncologically unresectable before treatment. Although patients who underwent surgery achieved better tumor response, response variables are not useful for prospective identification of potentially resectable patients at baseline. Therefore, we used this real-world dataset to develop criteria for identifying potentially resectable patients before initiation of combination therapy.

### Criteria for identifying potentially resectable advanced HCC before combination therapy

First, to ensure the safety of hepatectomy, patients should have an Eastern Cooperative Oncology Group performance status 0 or 1 and Child-Pugh class A. These criteria were met by all patients in the validation cohort who underwent surgery (**Table 1**).

To ensure R0 resection, we next proposed an ‘intrahepatic tumor criterion’ based on the number, location and/or size of intrahepatic tumors. To be considered potentially resectable, intrahepatic tumors should either be confined to a single lobe (left, right, or middle lobe), or be present in one lobe alongside a single tumor with diameter ≤5 cm or up to three tumors each with diameter ≤3 cm in the remaining lobes. Furthermore, R0 resection should be achievable with hemihepatectomy, alone or combined with locoregional therapy to the remaining lobes, such as local hepatectomy or radiofrequency ablation. To ensure a safe hepatectomy, FLRV should be >30% in non-cirrhotic patients and >40% cirrhotic patients. In our dataset, patients who met the intrahepatic tumor criterion had a significantly higher rate of surgery than those who did not (45.6% vs 2.3%, *P* < 0.001; **Supplementary Table 1**).

To assess whether patients with macrovascular invasion should be considered for conversion therapy, we compared clinicopathologic features and tumor responses between patients with or without this feature (**Table 2**). Although macrovascular invasion was not correlated with surgery in the overall population, patients with macrovascular invasion had higher hepatitis B virus DNA copy number, higher α-fetoprotein, higher protein induced by vitamin K absence or antagonist-II, larger intrahepatic tumor diameter, but similar tumor response than patients without macrovascular invasion. Although there was no significant difference in the tumor response and surgery rate between patients with and without macrovascular invasion, patients with macrovascular invasion had higher tumor burden at baseline, which may indicate the similar surgery rate between the two groups may be attributed to the different tumor burden. Therefore, if the two groups had similar tumor burden, patients with macrovascular invasion might achieve higher tumor response and higher surgery rate, which indicated that a subset of patients with macrovascular invasion are more likely to undergo surgery following conversion therapy. However, surgery was not associated with portal vein tumor thrombosis (PVTT) classification (Vp classification, *P* = 0.615; Cheng’s classification, *P* = 0.551) or hepatic vein tumor thrombosis (HVTT) classification (Vv classification, *P* = 0.728; **Supplementary Table 2**).

**Table 2:** Clinicopathologic features and response in patients with or without macrovascular invasion.

Variables	Without macrovascular invasion (n = 86)	With macrovascular invasion (n = 101)	*P* value
Age, years, mean ± standard deviation	55.66 ± 11.97	54.91 ± 10.94	0.657
Sex, n (%)			0.972
Female	10 (11.6)	13 (12.9)	
Male	76 (88.4)	88 (87.1)	
ECOG PS, n (%)			1
0–1	81 (94.2)	96 (95)	
2	5 (5.8)	5 (5)	
Child-Pugh class, n (%)			0.134
A	82 (95.3)	89 (88.1)	
B	4 (4.7)	12 (11.9)	
HBsAg, n (%)			0.201
Negative	19 (22.1)	14 (13.9)	
Positive	67 (77.9)	87 (86.1)	
HBV DNA, n (%)			<0.001
≤10^3^/mL	50 (58.1)	37 (36.6)	
>10^3^/mL	21 (24.4)	57 (56.4)	
N/A	15 (17.4)	7 (6.9)	
BCLC stage, n (%)			<0.001
B	37 (43)	0 (0)	
C	49 (57)	101 (100)	
CNLC stage, n (%)			<0.001
IIb	37 (43)	0 (0)	
IIIa	0 (0)	75 (74.3)	
IIIb	49 (57)	26 (25.7)	
Extrahepatic disease, n (%)			<0.001
No	37 (43)	75 (74.3)	
Yes	49 (57)	26 (25.7)	
AFP, ng/mL, median (IQR)	137.5 (7.2, 7503)	2541 (23.8, 24907)	0.009
AFP, n (%)			0.013
≤400 ng/mL	49 (57)	38 (37.6)	
>400 ng/mL	37 (43)	63 (62.4)	
PIVKA-II, mAU/mL, median (IQR)	1236.5 (95.75, 12255.75)	9014 (928, 39055)	<0.001
PIVKA-II, n (%)			0.005
≤1000 mAU/mL	40 (46.5)	26 (25.7)	
>1000 mAU/mL	46 (53.5)	75 (74.3)	
Diameter of intrahepatic tumors, median (IQR)	7.3 (2.71, 13.6)	11.6 (8, 15.1)	<0.001
Treatment line, n (%)			<0.001
1	61 (70.9)	92 (91.1)	
2	24 (27.9)	8 (7.9)	
3	1 (1.2)	1 (1)	
Anti-PD-1 antibody, n (%)			0.453
Camrelizumab	25 (29.1)	34 (33.7)	
Nivolumab	3 (3.5)	8 (7.9)	
Pembrolizumab	9 (10.5)	12 (11.9)	
Sintilimab	33 (38.4)	35 (34.7)	
Tislelizumab	6 (7)	7 (6.9)	
Toripalimab	10 (11.6)	5 (5)	
BOR per RECIST v1.1, n (%)			0.89
CR	2 (2.3)	2 (2)	
PR	22 (25.6)	23 (22.8)	
SD	41 (47.7)	46 (45.5)	
PD	21 (24.4)	30 (29.7)	
Objective response per RECIST v1.1, n (%)			0.747
No	62 (72.1)	76 (75.2)	
Yes	24 (27.9)	25 (24.8)	
Disease control per RECIST v1.1, n (%)			0.52
No	21 (24.4)	30 (29.7)	
Yes	65 (75.6)	71 (70.3)	
BOR per mRECIST, n (%)			0.858
CR	4 (4.7)	5 (5)	
PR	30 (34.9)	31 (30.7)	
SD	31 (36)	35 (34.7)	
PD	21 (24.4)	30 (29.7)	
Objective response per mRECIST, n (%)			0.692
No	52 (60.5)	65 (64.4)	
Yes	34 (39.5)	36 (35.6)	
Disease control per mRECIST, n (%)			0.52
No	21 (24.4)	30 (29.7)	
Yes	65 (75.6)	71 (70.3)	
Change from baseline per RECIST v1.1, median (IQR)	-0.17 (-0.33, 0)	-0.13 (-0.29, 0)	0.399
Change from baseline per mRECIST, median (IQR)	-0.3 (-0.7, 0)	-0.3 (-0.54, 0)	0.515
Surgery after therapy, n (%)			0.2
No	69 (80.2)	89 (88.1)	
Yes	17 (19.8)	12 (11.9)	

AFP, a-fetoprotein; BCLC, Barcelona Clinic liver cancer; BOR, best overall response; CNLC, China liver cancer; CR, complete response; DCP, des-gamma-carboxy prothrombin; ECOG PS, Eastern Cooperative Oncology Group performance status; HBsAg, hepatitis B surface antigen; HBV, hepatitis B virus; IQR, interquartile range; mRECIST, modified Response Evaluation Criteria in Solid Tumors; PD-1, programmed death-1; PD, progressive disease; PIVKA-II, protein induced by vitamin K absence or antagonist-II; PR, partial response; RECIST, Response Evaluation Criteria in Solid Tumors; SD, stable disease.

Therefore, we proposed the following tumor thrombosis criterion which might help identify potentially resectable patients with macrovascular invasion: PVTT should not involve the contralateral liver lobe and not reach the superior mesenteric vein, while HVTT should involve no more than two major hepatic vein branches on the tumor side, and any tumor thrombus of the inferior vena cava should not reach the atrium. Patients without tumor thrombosis are considered to meet this criterion. Patients who met the tumor thrombosis criterion had a higher surgery rate than patients who did not (17.5% vs 0%, *P* = 0.049; **Supplementary Table 3**). Furthermore, sensitivity analysis in patients with macrovascular invasion showed that meeting the tumor thrombosis criterion was associated with a marginally higher surgery rate in this patient subgroup (15.0% vs 0%, *P* = 0.067; **Supplementary Table 4**).

Since patients with and without extrahepatic metastases had a similar probability of undergoing surgery (13.3% vs 17.0%, respectively; *P* = 0.641; **Supplementary Table 5**), patients with extrahepatic metastases at baseline were not excluded from the potentially resectable population.

The proposed comprehensive criteria for identifying initially oncologically unresectable patients who are potentially resectable before initiating combination therapy are summarized in **Table 3**. The criteria define the upper limit of the potentially resectable population, beyond which the population is less likely to achieve successful conversion. Patients who meet the criteria are more likely, but not guaranteed, to achieve resectability.

**Table 3:** Proposed criteria for patients with potentially resectable HCC before lenvatinib plus anti-PD-1 therapy.

Factor	Criterion
General condition	ECOG PS: 0–1
Liver function reserve	Child-Pugh class: A
Intrahepatic tumors	Tumors confined to one lobe (left, right, or middle lobe), or tumors in one lobe are present alongside a single tumor with diameter ≤5 cm or up to three tumors each with diameter ≤3 cm in the remaining lobes. R0 resection can be achieved with hemihepatectomy, alone or combined with locoregional therapy, such as local hepatectomy or radiofrequency ablation, to the remaining lobes during surgery*
Macrovascular invasion^†^	No PVTT involving the contralateral liver lobe or reaching the superior mesenteric vein. No HVTT involving more than two major hepatic vein branches on the tumor side, and no tumor thrombus of the inferior vena cava reaching the atrium

^*^To ensure a safe R0 resection, the FLRV should be >30% in non-cirrhotic and >40% in cirrhotic patients.

^†^Patients without a tumor thrombosis are considered to meet this criterion.

ECOG, PS Eastern Cooperative Oncology Group performance status; FLRV, future liver remnant volume; HCC, hepatocellular carcinoma; HVTT, hepatic vein tumor thrombus; PD-1, programmed death-1; PVTT, portal vein tumor thrombus.

### Proposed criteria as an independent predictor of surgery

A comparison of clinicopathologic features and tumor responses between patients who did (i.e. potentially resectable patients) and those who did not meet the proposed criteria before combination therapy with lenvatinib plus an anti-PD-1 antibody is summarized in **Table 4**. Of 187 patients, 56 (29.9%) met the criteria and were therefore considered potentially resectable. Patients who met the criteria had better liver function, earlier treatment lines, better tumor response, and higher surgery rate (46.4% vs 2.3%, *P* < 0.001) than those who did not meet the criteria. Furthermore, multivariate Firth logistic regression confirmed that meeting the criteria was an independent predictor of surgery, whether the multivariate model included a covariate for objective response per RECIST v1.1 (odds ratio [OR], 31.613; 95% CI, 10.119–136.382, *P* < 0.001) or mRECIST (OR, 28.826; 95% CI, 8.783–131.873, *P* < 0.001) (**Table 5**).

**Table 4:** Clinicopathologic features and response according to attainment of potentially resectable criteria.

Variables	Potentially resectable criteria not met (n = 131)	Potentially resectable criteria met (n = 56)	*P* value
Age, years, mean ± standard deviation	54.66 ± 11.46	56.66 ± 11.24	0.269
Sex, n (%)			0.5
Female	18 (13.7)	5 (8.9)	
Male	113 (86.3)	51 (91.1)	
ECOG PS, n (%)			0.726
0–1	123 (93.9)	54 (96.4)	
2	8 (6.1)	2 (3.6)	
Child-Pugh class, n (%)			0.042
A	116 (88.5)	55 (98.2)	
B	15 (11.5)	1 (1.8)	
HBsAg, n (%)			0.796
Negative	22 (16.8)	11 (19.6)	
Positive	109 (83.2)	45 (80.4)	
HBV DNA, n (%)			0.620
≤10^3^/mL	56 (42.7)	31 (55.4)	
>10^3^/mL	54 (41.2)	24 (42.9)	
N/A	21 (16)	1 (1.8)	
BCLC stage, n (%)			0.332
B	23 (17.6)	14 (25)	
C	108 (82.4)	42 (75)	
CNLC stage, n (%)			0.473
IIb	23 (17.6)	14 (25)	
IIIa	55 (42)	20 (35.7)	
IIIb	53 (40.5)	22 (39.3)	
Extrahepatic disease, n (%)			1
No	78 (59.5)	34 (60.7)	
Yes	53 (40.5)	22 (39.3)	
Macrovascular invasion, n (%)			0.23
No	56 (42.7)	30 (53.6)	
Yes	75 (57.3)	26 (46.4)	
AFP, ng/mL, median (IQR)	1187 (16.65, 16062.5)	236.5 (7.33, 12954)	0.166
AFP, n (%)			0.081
≤400 ng/mL	55 (42)	32 (57.1)	
>400 ng/mL	76 (58)	24 (42.9)	
PIVKA-II, mAU/mL, median (IQR)	3827 (302, 26711)	3363 (194.25, 19566.5)	0.428
PIVKA-II, n (%)			0.562
≤1000 mAU/mL	44 (33.6)	22 (39.3)	
>1000 mAU/mL	87 (66.4)	34 (60.7)	
Diameter of intrahepatic tumors, median (IQR)	10 (4.74, 14.65)	9.15 (5.3, 14.22)	0.661
Treatment line, n (%)			0.021
1	101 (77.1)	52 (92.9)	
2	28 (21.4)	4 (7.1)	
3	2 (1.5)	0 (0)	
Anti-PD-1 antibody, n (%)			0.672
Camrelizumab	39 (29.8)	20 (35.7)	
Nivolumab	10 (7.6)	1 (1.8)	
Pembrolizumab	15 (11.5)	6 (10.7)	
Sintilimab	48 (36.6)	20 (35.7)	
Tislelizumab	8 (6.1)	5 (8.9)	
Toripalimab	11 (8.4)	4 (7.1)	
BOR per RECIST v1.1, n (%)			<0.001
CR	1 (0.8)	3 (5.4)	
PR	27 (20.6)	18 (32.1)	
SD	56 (42.7)	31 (55.4)	
PD	47 (35.9)	4 (7.1)	
Objective response per RECIST v1.1, n (%)			0.034
No	103 (78.6)	35 (62.5)	
Yes	28 (21.4)	21 (37.5)	
Disease control per RECIST v1.1, n (%)			<0.001
No	47 (35.9)	4 (7.1)	
Yes	84 (64.1)	52 (92.9)	
BOR per mRECIST, n (%)			<0.001
CR	5 (3.8)	4 (7.1)	
PR	32 (24.4)	29 (51.8)	
SD	47 (35.9)	19 (33.9)	
PD	47 (35.9)	4 (7.1)	
Objective response per mRECIST, n (%)			<0.001
No	94 (71.8)	23 (41.1)	
Yes	37 (28.2)	33 (58.9)	
Disease control per mRECIST, n (%)			<0.001
No	47 (35.9)	4 (7.1)	
Yes	84 (64.1)	52 (92.9)	
Change from baseline per RECIST v1.1, median (IQR)	-0.13 (-0.32, 0)	-0.21 (-0.35, -0.02)	0.081
Change from baseline per mRECIST, median (IQR)	-0.16 (-0.46, 0)	-0.45 (-0.78, -0.09)	0.002
Surgery after therapy, n (%)			<0.001
No	128 (97.7)	30 (53.6)	
Yes	3 (2.3)	26 (46.4)	

AFP, a-fetoprotein; BCLC, Barcelona Clinic liver cancer; BOR, best overall response; CNLC, China liver cancer; CR, complete response; ECOG, PS Eastern Cooperative Oncology Group performance status; HBsAg, hepatitis B surface antigen; HBV, hepatitis B virus; IQR, interquartile range; mRECIST, modified Response Evaluation Criteria in Solid Tumors; N/A, not available; PD-1, programmed death-1; PD, progressive disease; PIVKA-II, protein induced by vitamin K absence or antagonist-II; PR, partial response; RECIST, Response Evaluation Criteria in Solid Tumors; SD, stable disease.

**Table 5:** Univariate and multivariate Firth logistic regression analysis for predictors of surgery.

	Univariate analysis			Multivariate analysisincluding response per RECIST v1.1			Multivariate analysisincluding response per mRECIST			
Variable	OR	95% CI	*P* value	OR	95% CI	*P* value	OR	95% CI	*P* value
Age									
≥56 vs <56 years	0.386	0.156–0.877	0.022	0.415	0.14–1.151	0.091	0.418	0.134–1.211	0.109
Sex									
Male vs female	1.121	0.371–4.45	0.852						
ECOG PS									
1 vs 0	0.612	0.223–1.479	0.286						
2 vs 0	0.24	0.002–1.954	0.226						
Child-Pugh class									
B vs A	0.146	0.001–1.138	0.072	0.137	0.001–2.674	0.211	0.153	0.001–2.867	0.233
HbsAg									
Positive vs negative	0.973	0.375–2.93	0.957						
HBV DNA									
>10^3^/mL vs ≤10^3^/mL	0.893	0.399–1.973	0.78						
BCLC stage*									
C vs B	0.471	0.201–1.163	0.1						
CNLC stage									
IIIa vs II;b	0.769	0.33–1.706	0.523						
IIIb vs II;b	0.769	0.33–1.706	0.523						
Extrahepatic disease									
Yes vs no	0.769	0.33–1.706	0.523	0.286	0.076–0.911	0.034	0.239	0.056–0.831	0.023
Macrovascular invasion									
Yes vs no	0.555	0.247–1.215	0.141	0.651	0.219–1.894	0.43	0.588	0.182–1.828	0.358
AFP									
> vs ≤ 400 ng/mL	0.784	0.356–1.718	0.542						
PIVKA-II									
> vs ≤ 1000 mAU/mL	0.863	0.39–1.976	0.72						
Treatment line									
2 vs 1	0.383	0.075–1.258	0.122	0.585	0.086–3.19	0.543	0.635	0.067–4.186	0.653
3 vs 1	1.061	0.008–13.48	0.97	12.956	0.084–243.306	0.226	24.542	0.152–552.914	0.156
Objective response per RECIST v1.1									
Yes vs no	3.858	1.717–8.758	0.001	5.11	1.695–17.382	0.003			
Objective response per mRECIST									
Yes vs no	8.487	3.514–23.309	<0.001				9.031	2.892–34.909	<0.001
Potentially resectable criteria met									
Yes vs no	31.899	10.971–124.71	<0.001	31.613	10.119–136.382	<0.001	28.826	8.783–131.873	<0.001

*As patients with BCLC stage C consist of those with macrovascular invasion or extrahepatic metastasis, macrovascular invasion and extrahepatic metastasis were included individually in the multivariate analysis instead of BCLC stage (C vs. B).

AFP, a-fetoprotein; BCLC, Barcelona Clinic liver cancer; CI, confidence interval; CNLC, China liver cancer; ECOG PS, Eastern Cooperative Oncology Group performance status; HbsAg, hepatitis B surface antigen; HBV, hepatitis B virus; mRECIST, modified Response Evaluation Criteria in Solid Tumors; OR, odds ratio; PIVKA-II, protein induced by vitamin K absence or antagonist-II; RECIST, Response Evaluation Criteria in Solid Tumors.

## Discussion

In this study, we proposed criteria for identifying potentially resectable patients with initially oncologically unresectable HCC based on real-world evidence from a cohort of patients who received lenvatinib plus an anti-PD-1 antibody. We further confirmed our criteria were independent predictors of surgery in this cohort. To our knowledge, this is the first study to define clearly the clinicopathologic features of potentially resectable patients with initially oncologically unresectable HCC.

Although conversion therapy with systemic therapy, with or without locoregional therapy, is a feasible strategy for patients with initially unresectable or advanced HCC, prior studies included different populations and did not clearly define criteria for potential resectability and successful downstaging, leading to variability in reported conversion rates and outcomes (**Table 6**). Furthermore, while conversion therapy is feasible, the successful conversion rate is generally low. Therefore, identification of the population most likely to benefit from a conversion strategy is an important clinical priority. For oncologically unresectable patients, both suitable tumor burden and effective preoperative anti-tumor therapy are prerequisites for successful conversion. Since ORRs with currently available systemic therapy for advanced HCC (30–40%) are unlikely to be improved in the short term, identification of patients with appropriate tumor burden (i.e. potentially resectable patients) is a key strategy for improving the successful conversion rate.

**Table 6:** Studies reporting surgery rates following systemic (± locoregional) therapy in initially unresectable HCC.

Reference	Study design	PVTT	Extrahepatic disease	Downstaging treatment (sample size)	ORR per RECIST v1.1	Surgery rate	Survival outcome
He et al., 2019 (9)	Multicenter, randomized, open-label	Yes	Yes	Sorafenib plus HAIC (125) vs sorafenib (122)	40.8% vs 2.5%	12.8% vs 0.8%	Median OS: 13.37 mo vs 7.13 mo Median PFS: 7.03 mo vs 2.6 mo
He et al., 2021 (10)	Multicenter, retrospective	Yes	Yes	Lenvatinib + anti-PD-1 + HAIC (71) vs lenvatinib (86)	59.2% vs 9.3%	12.7% vs 0%	Median OS: NR vs 11.0 mo Median PFS: 11.1 mo vs 5.1 mo
Zhu et al., 2021 (11)	Single-center, retrospective	Yes	Yes	TKI + anti-PD-1 (63)	N/A	15.9%	N/A
Shindoh et al., 2021 (6)	Single-center, retrospective	Yes	Yes	Lenvatinib (107)	36.4%	11.2%	Median OS for R0 and R2 resection: 19.0 mo and 8.9 mo, respectively
Ho et al., 2021 (12)	Single-center, single-arm, open-label	Yes	No	Cabozantinib plus nivolumab (15)	7%	80%	DFS >7.8 mo for patients with pathological responses 1.9 mo < DFS < 5.2 mo for patients without pathological responses
Yang et al., 2021 (13)	Single-center, retrospective	N/A	Yes	TKI + anti-PD-1 + locoregional therapy (38)	55.6% (mRECIST)	23.7%	N/A
Zhang et al., 2021 (14)	Single-center, retrospective	Yes	No	TKI + anti-PD-1 (N/A)	N/A	N/A (eight patients underwent surgery)	12-mo OS rate: 75% 12-mo RFS rate: 75%
Zhang et al., 2021 (15)	Single-center, retrospective	Yes	Yes	TKI + PD-1 + HAIC (25)	84%	56%	Median OS/PFS: NR after a median follow-up of 12.53 mo

DFS, disease-free survival; HAIC, hepatic arterial infusion chemotherapy; HCC, hepatocellular carcinoma; mo, months; mRECIST, modified Response Evaluation Criteria in Solid Tumors; N/A, not available; NR, not reached; ORR, objective response rate; OS, overall survival; PD-1, programmed death-1; PFS, progression-free survival; PVTT, portal vein tumor thrombus; RECIST, Response Evaluation Criteria in Solid Tumors; RFS, recurrence-free survival; TKI, tyrosine kinase inhibitor.

Our proposed criteria might define the upper limit of the potentially resectable population. Patients who met the criteria were significantly more likely to undergo surgery following combination therapy with lenvatinib plus an anti-PD-1 antibody, and meeting the criteria was an independent predictor of surgery, with an odds ratio of ~30 (**Table 5**). Objective response was also an independent predictor for conversion in the multivariate analysis, underscoring the importance of achieving objective response or greater depth of tumor response for successful conversion. For patients who do not meet our criteria, alternative treatment strategies with higher ORRs, such as systemic therapy plus locoregional therapy, should be considered to improve the probability of resection. However, as patients who do not meet our criteria may have poorer liver function, greater tumor burden and poorer tolerance to anti-tumor treatment, the safety of combination of systemic therapy plus locoregional therapy must be considered. Multivariate analysis also indicated that patients with extrahepatic metastases were less likely to undergo surgery than those without extrahepatic metastases, with ORs in the range of 0.2–0.3. This result is expected given that patients with extrahepatic metastases require anti-tumor efficacy not only in intrahepatic tumors, but also in extrahepatic lesions. In our study, 22 of 75 patients (29.3%) with extrahepatic metastases met the proposed criteria (which do not themselves account for extrahepatic metastases). Of these, 9 (40.9%) underwent surgery, which is a slightly lower proportion compared with patients without extrahepatic metastases (17 of 34, 50.0%).

Patients with initially oncologically unresectable HCC may achieve successful conversion after systemic therapy, but some of them might not receive conversion surgery due to some reasons. In this study, two initially oncologically unresectable patients, who met the potentially resectable criteria at baseline, achieved successful conversion after combination therapy, but they refused to undergo surgical resection for personal reasons. One patient was 83 years old; he and his family refused surgery because of his advanced age. The other patient refused surgery for financial reasons. If these two patients underwent conversion surgery, patients who met the proposed potentially resectable criteria would have a higher surgery rate of 50.0% (28 of 56), which was 46.4% (26 of 56) in the real-world situation (**Table 4**).

In addition to supporting the identification of potentially resectable patients, adoption of the proposed criteria could also facilitate comparisons between future studies of conversion therapy for HCC. The criteria could serve as a basis for patient inclusion in clinical trials investigating conversion therapy in this setting.

Study limitations include the use of a single-center, retrospective cohort with a modest sample size for validation of the proposed criteria. Prospective, multicenter data are warranted for further validation. In addition, our cohort included only patients who received combination therapy with lenvatinib plus an anti-PD-1 antibody. Further studies including patients treated with different conversion regimens are required.

In conclusion, we proposed and validated criteria for identifying patients with initially oncologically unresectable HCC who are potentially resectable following combination therapy with lenvatinib plus an anti-PD-1 antibody. The proposed criteria could be used to standardize conversion therapy research in advanced HCC.

## Data availability statement

The original contributions presented in the study are included in the article/**Supplementary Material**. Further inquiries can be directed to the corresponding authors.

## Ethics statement

This study involving human participants was reviewed and approved by Zhongshan Hospital Research Ethics Committee (Approval Number: B2020-177R). The patients/participants provided their written informed consent before initiating combination therapy.

## Author contributions

Study concept and design (BX, XD-Z, YH-S, HC-S, and CH); acquisition of data (BX, XD-Z, YH-S, JJ-Z, JL, ML-L, PW-T, HC-S, and CH); analysis and interpretation of data (all authors); drafting of the manuscript (BX, HC-S, and CH); critical revision of the manuscript for important intellectual content (all authors); statistical analysis (BX, XD-Z, YH-S, HC-S, and CH); obtained funding (HC-S, and CH); administrative, technical, or material support, study supervision (JZ, JF, HC-S, and CH). All authors read and approved the final manuscript.

## Funding

This work was supported by the Leading Investigator Program of the Shanghai municipal government (17XD1401100) to HC-S, the National Key Basic Research Program (973 Program, 2015CB554005) from the Ministry of Science and Technology of China to HC-S, the National Natural Science Foundation of China (81871928 to HC-S, 81871929 and 82072667 to CH), and the Special Research Fund for Liver Cancer Diagnosis and Treatment from the China Anti-Cancer Association (H2020-008 to HC-S, H2020-044 to CH). This study received funding from Eisai China lnc. The funder was not involved in the study design, collection, analysis, interpretation of data, the writing of this article or the decision to submit it for publication.

## Acknowledgments

Editorial support was provided by Mark Dyson, DPhil (Berlin, Germany) on behalf of Rude Health Consulting and funded by Eisai China lnc.

## Conflict of interest

HC-S has received honorarium or lecture fees from Roche, Bayer, MSD, Eisai, Hengrui, Innovent, TopAlliance, Abbott, Beigene, Gilead, and Zelgen during the last 5 years.

The remaining authors declare that the research was conducted in the absence of any commercial or financial relationships that could be construed as a potential conflict of interest.

## Publisher’s note

All claims expressed in this article are solely those of the authors and do not necessarily represent those of their affiliated organizations, or those of the publisher, the editors and the reviewers. Any product that may be evaluated in this article, or claim that may be made by its manufacturer, is not guaranteed or endorsed by the publisher.
