# Supplementary material for: Criteria for identifying potentially resectable patients with initially oncologically unresectable hepatocellular carcinoma before treatment with lenvatinib plus an anti–PD–1 antibody
Source: Front Immunol. 2022 Nov 25;13:1016736. doi: 10.3389/fimmu.2022.1016736 (PMC9732007; doi:10.3389/fimmu.2022.1016736)
Supplement: Supplementary file 1 [file Table_1.docx]

Supplementary Material

# Supplementary Tables

**Supplementary Table 1. Comparison of clinicopathologic features and tumor responses between patients who did or did not meet the intrahepatic tumor criterion**

| **Variables** | **Intrahepatic tumor criterion not met**  **(n = 130)** | **Intrahepatic tumor criterion met**  **(n = 57)** | ***P* value** |
| --- | --- | --- | --- |
| Age, years, mean ± standard deviation | 54.68 ± 11.5 | 56.56 ± 11.16 | 0.297 |
| Sex, n (%) |  |  | 0.465 |
| Female | 18 (13.8) | 5 (8.8) |  |
| Male | 112 (86.2) | 52 (91.2) |  |
| ECOG PS, n (%) |  |  | 0.726 |
| 0–1 | 122 (93.8) | 55 (96.5) |  |
| 2 | 8 (6.2) | 2 (3.5) |  |
| Child-Pugh class, n (%) |  |  | **0.043** |
| A | 115 (88.5) | 56 (98.2) |  |
| B | 15 (11.5) | 1 (1.8) |  |
| HBsAg, n (%) |  |  | 0.854 |
| Negative | 22 (16.9) | 11 (19.3) |  |
| Positive | 108 (83.1) | 46 (80.7) |  |
| HBV DNA, n (%) |  |  | 0.749 |
| ≤10^3^/mL | 56 (43.1) | 31 (54.4) |  |
| >10^3^/mL | 53 (40.8) | 25 (43.9) |  |
| N/A | 21 (16.2) | 1 (1.8) |  |
| BCLC stage, n (%) |  |  | 0.376 |
| B | 23 (17.7) | 14 (24.6) |  |
| C | 107 (82.3) | 43 (75.4) |  |
| CNLC stage, n (%) |  |  | 0.482 |
| IIb | 23 (17.7) | 14 (24.6) |  |
| IIIa | 55 (42.3) | 20 (35.1) |  |
| IIIb | 52 (40) | 23 (40.4) |  |
| Extrahepatic disease, n (%) |  |  | 1 |
| No | 78 (60) | 34 (59.6) |  |
| Yes | 52 (40) | 23 (40.4) |  |
| Macrovascular invasion, n (%) |  |  | 0.295 |
| No | 56 (43.1) | 30 (52.6) |  |
| Yes | 74 (56.9) | 27 (47.4) |  |
| AFP, ng/mL, median (IQR) | 1145.5 (15.03, 15785.5) | 245 (7.4, 15495) | 0.226 |
| AFP, n (%) |  |  | 0.113 |
| ≤400 ng/mL | 55 (42.3) | 32 (56.1) |  |
| >400 ng/mL | 75 (57.7) | 25 (43.9) |  |
| PIVKA-II, mAU/mL, median (IQR) | 3762 (294, 26918) | 3468 (195, 18699) | 0.448 |
| PIVKA-II, n (%) |  |  | 0.646 |
| ≤1000 mAU/mL | 44 (33.8) | 22 (38.6) |  |
| >1000 mAU/mL | 86 (66.2) | 35 (61.4) |  |
| Diameter of intrahepatic tumors, median (IQR) | 9.95 (4.67, 14.67) | 9.3 (5.4, 14.2) | 0.681 |
| Treatment line, n (%) |  |  | **0.019** |
| 1 | 100 (76.9) | 53 (93) |  |
| 2 | 28 (21.5) | 4 (7) |  |
| 3 | 2 (1.5) | 0 (0) |  |
| Anti-PD-1 antibody used, n (%) |  |  | 0.68 |
| Camrelizumab | 39 (30) | 20 (35.1) |  |
| Nivolumab | 10 (7.7) | 1 (1.8) |  |
| Pembrolizumab | 15 (11.5) | 6 (10.5) |  |
| Sintilimab | 47 (36.2) | 21 (36.8) |  |
| Tislelizumab | 8 (6.2) | 5 (8.8) |  |
| Toripalimab | 11 (8.5) | 4 (7) |  |
| BOR per RECIST v1.1, n (%) |  |  | **<0.001** |
| CR | 1 (0.8) | 3 (5.3) |  |
| PR | 27 (20.8) | 18 (31.6) |  |
| SD | 56 (43.1) | 31 (54.4) |  |
| PD | 46 (35.4) | 5 (8.8) |  |
| Objective response per RECIST v1.1, n (%) |  |  | **0.044** |
| No | 102 (78.5) | 36 (63.2) |  |
| Yes | 28 (21.5) | 21 (36.8) |  |
| Disease control per RECIST v1.1, n (%) |  |  | **<0.001** |
| No | 46 (35.4) | 5 (8.8) |  |
| Yes | 84 (64.6) | 52 (91.2) |  |
| BOR per mRECIST, n (%) |  |  | **<0.001** |
| CR | 5 (3.8) | 4 (7) |  |
| PR | 32 (24.6) | 29 (50.9) |  |
| SD | 47 (36.2) | 19 (33.3) |  |
| PD | 46 (35.4) | 5 (8.8) |  |
| Objective response per mRECIST, n (%) |  |  | **<0.001** |
| No | 93 (71.5) | 24 (42.1) |  |
| Yes | 37 (28.5) | 33 (57.9) |  |
| Disease control per mRECIST, n (%) |  |  | **<0.001** |
| No | 46 (35.4) | 5 (8.8) |  |
| Yes | 84 (64.6) | 52 (91.2) |  |
| Change from baseline per RECIST v1.1, median (IQR) | -0.13 (-0.32, 0) | -0.21 (-0.35, -0.02) | 0.081 |
| Change from baseline per mRECIST, median (IQR) | -0.16 (-0.46, 0) | -0.45 (-0.78, -0.09) | **0.002** |
| Surgery after therapy, n (%) |  |  | **<0.001** |
| No | 127 (97.7) | 31 (54.4) |  |
| Yes | 3 (2.3) | 26 (45.6) |  |

*AFP* α-fetoprotein, *BCLC* Barcelona Clinic liver cancer, *BOR* best overall response, *CNLC* China liver cancer, *CR* complete response, *ECOG PS* Eastern Cooperative Oncology Group performance status, *HBsAg* hepatitis B surface antigen, *HBV* hepatitis B virus, *IQR* interquartile range, *mRECIST* modified Response Evaluation Criteria in Solid Tumors, *N/A*, not available, *PD-1* programmed death-1, *PD* progressive disease, PIVKA-II protein induced by vitamin K absence or antagonist-II, *PR* partial response, *RECIST* Response Evaluation Criteria in Solid Tumors, *SD* stable disease.

**Supplementary Table 2. Association between PVTT or HVTT classification and surgery after therapy**

| **Variables** | **No surgery**  **(n = 158)** | **Surgery**  **(n = 29)** | ***P* value** |
| --- | --- | --- | --- |
| Vp classification, n (%) |  |  | 0.615 |
| No PVTT | 80 (50.6) | 18 (62.1) |  |
| Vp1 | 0 (0) | 0 (0) |  |
| Vp2 | 8 (5.1) | 2 (6.9) |  |
| Vp3 | 28 (17.7) | 3 (10.3) |  |
| Vp4 | 42 (26.6) | 6 (20.7) |  |
| Cheng’s classification, n (%) |  |  | 0.551 |
| No PVTT | 80 (50.6) | 18 (62.1) |  |
| Ⅰ | 8 (5.1) | 2 (6.9) |  |
| Ⅱ | 28 (17.7) | 3 (10.3) |  |
| Ⅲ | 39 (24.7) | 5 (17.2) |  |
| Ⅳ | 3 (1.9) | 1 (3.4) |  |
| Vv classification, n (%) |  |  | 0.728 |
| No HVTT | 135 (85.4) | 27 (93.1) |  |
| Vv1 | 0 (0) | 0 (0) |  |
| Vv2 | 8 (5.1) | 1 (3.4) |  |
| Vv3 | 15 (9.5) | 1 (3.4) |  |

*HVTT* hepatic vein tumor thrombosis, *PVTT* portal vein tumor thrombosis.

**Supplementary Table 3. Comparison of clinicopathologic features and tumor responses between patients who did or did** **not meet the tumor thrombosis criterion**

| **Variables** | **Tumor thrombosis criterion not met**  **(n = 21)** | **Tumor thrombosis criterion met**  **(n = 166)** | ***P* value** |
| --- | --- | --- | --- |
| Age, years, mean ± standard deviation | 51.38 ± 10.92 | 55.75 ± 11.4 | 0.098 |
| Sex, n (%) |  |  | 1 |
| Female | 2 (9.5) | 21 (12.7) |  |
| Male | 19 (90.5) | 145 (87.3) |  |
| ECOG PS, n (%) |  |  | 0.606 |
| 0–1 | 21 (100) | 156 (94) |  |
| 2 | 0 (0) | 10 (6) |  |
| Child-Pugh class, n (%) |  |  | 0.397 |
| A | 18 (85.7) | 153 (92.2) |  |
| B | 3 (14.3) | 13 (7.8) |  |
| HBsAg, n (%) |  |  | 0.769 |
| Negative | 4 (19) | 29 (17.5) |  |
| Positive | 17 (81) | 137 (82.5) |  |
| HBV DNA, n (%) |  |  | 0.996 |
| ≤10^3^/mL | 10 (47.6) | 77 (46.4) |  |
| >10^3^/mL | 8 (38.1) | 70 (42.2) |  |
| N/A | 3 (14.3) | 19 (11.4) |  |
| BCLC stage, n (%) |  |  | **0.016** |
| B | 0 (0) | 37 (22.3) |  |
| C | 21 (100) | 129 (77.7) |  |
| CNLC stage, n (%) |  |  | **<0.001** |
| IIb | 0 (0) | 37 (22.3) |  |
| IIIa | 17 (81) | 58 (34.9) |  |
| IIIb | 4 (19) | 71 (42.8) |  |
| Extrahepatic disease, n (%) |  |  | 0.064 |
| No | 17 (81) | 95 (57.2) |  |
| Yes | 4 (19) | 71 (42.8) |  |
| Macrovascular invasion, n (%) |  |  | **<0.001** |
| No | 0 (0) | 86 (51.8) |  |
| Yes | 21 (100) | 80 (48.2) |  |
| AFP, ng/mL, median (IQR) | 2956 (114, 7585) | 495.4 (10.25, 16148.75) | 0.37 |
| AFP, n (%) |  |  | 0.292 |
| ≤400 ng/mL | 7 (33.3) | 80 (48.2) |  |
| >400 ng/mL | 14 (66.7) | 86 (51.8) |  |
| PIVKA-II, mAU/mL, median (IQR) | 15970 (1029, 55532) | 3298.5 (252.25, 23066.75) | 0.145 |
| PIVKA-II, n (%) |  |  | 0.354 |
| ≤1000 mAU/mL | 5 (23.8) | 61 (36.7) |  |
| >1000 mAU/mL | 16 (76.2) | 105 (63.3) |  |
| Diameter of intrahepatic tumors, median (IQR) | 11.6 (7.7, 15.1) | 9.35 (4.83, 14.28) | 0.162 |
| Treatment line, n (%) |  |  | 0.317 |
| 1 | 20 (95.2) | 133 (80.1) |  |
| 2 | 1 (4.8) | 31 (18.7) |  |
| 3 | 0 (0) | 2 (1.2) |  |
| Anti-PD-1 antibody used, n (%) |  |  | 0.973 |
| Camrelizumab | 8 (38.1) | 51 (30.7) |  |
| Nivolumab | 1 (4.8) | 10 (6) |  |
| Pembrolizumab | 3 (14.3) | 18 (10.8) |  |
| Sintilimab | 7 (33.3) | 61 (36.7) |  |
| Tislelizumab | 1 (4.8) | 12 (7.2) |  |
| Toripalimab | 1 (4.8) | 14 (8.4) |  |
| BOR per RECIST v1.1, n (%) |  |  | 0.434 |
| CR | 0 (0) | 4 (2.4) |  |
| PR | 4 (19) | 41 (24.7) |  |
| SD | 8 (38.1) | 79 (47.6) |  |
| PD | 9 (42.9) | 42 (25.3) |  |
| Objective response per RECIST v1.1, n (%) |  |  | 0.597 |
| No | 17 (81) | 121 (72.9) |  |
| Yes | 4 (19) | 45 (27.1) |  |
| Disease control per RECIST v1.1, n (%) |  |  | 0.149 |
| No | 9 (42.9) | 42 (25.3) |  |
| Yes | 12 (57.1) | 124 (74.7) |  |
| BOR per mRECIST, n (%) |  |  | 0.378 |
| CR | 1 (4.8) | 8 (4.8) |  |
| PR | 5 (23.8) | 56 (33.7) |  |
| SD | 6 (28.6) | 60 (36.1) |  |
| PD | 9 (42.9) | 42 (25.3) |  |
| Objective response per mRECIST, n (%) |  |  | 0.515 |
| No | 15 (71.4) | 102 (61.4) |  |
| Yes | 6 (28.6) | 64 (38.6) |  |
| Disease control per mRECIST, n (%) |  |  | 0.149 |
| No | 9 (42.9) | 42 (25.3) |  |
| Yes | 12 (57.1) | 124 (74.7) |  |
| Change from baseline per RECIST v1.1, median (IQR) | -0.16 (-0.19, -0.02) | -0.15 (-0.33, 0) | 0.931 |
| Change from baseline per mRECIST, median (IQR) | -0.32 (-0.63, -0.09) | -0.3 (-0.63, 0) | 0.557 |
| Surgery after therapy, n (%) |  |  | **0.049** |
| No | 21 (100) | 137 (82.5) |  |
| Yes | 0 (0) | 29 (17.5) |  |

*AFP* α-fetoprotein, *BCLC* Barcelona Clinic liver cancer, *BOR* best overall response, *CNLC* China liver cancer, *CR* complete response, *ECOG PS* Eastern Cooperative Oncology Group performance status, *HBsAg* hepatitis B surface antigen, *HBV* hepatitis B virus, *IQR* interquartile range, *mRECIST* modified Response Evaluation Criteria in Solid Tumors, *N/A*, not available, *PD-1* programmed death-1, *PD* progressive disease, PIVKA-II protein induced by vitamin K absence or antagonist-II, *PR* partial response, *RECIST* Response Evaluation Criteria in Solid Tumors, *SD* stable disease.

**Supplementary Table 4. Comparison of clinicopathologic features and tumor responses between patients with macrovascular invasion who did or did not meet the tumor thrombosis criterion**

| **Variables** | **Tumor thrombosis criterion not met**  **(n = 21)** | **Tumor thrombosis criterion met**  **(n = 80)** | ***P* value** |
| --- | --- | --- | --- |
| Age, years, mean ± standard deviation | 51.38 ± 10.92 | 55.84 ± 10.82 | 0.105 |
| Sex, n (%) |  |  | 1 |
| Female | 2 (9.5) | 11 (13.8) |  |
| Male | 19 (90.5) | 69 (86.2) |  |
| ECOG PS, n (%) |  |  | 0.581 |
| 0–1 | 21 (100) | 75 (93.8) |  |
| 2 | 0 (0) | 5 (6.2) |  |
| Child-Pugh class, n (%) |  |  | 0.71 |
| A | 18 (85.7) | 71 (88.8) |  |
| B | 3 (14.3) | 9 (11.2) |  |
| HBsAg, n (%) |  |  | 0.482 |
| Negative | 4 (19) | 10 (12.5) |  |
| Positive | 17 (81) | 70 (87.5) |  |
| HBV DNA, n (%) |  |  | 0.195 |
| ≤10^3^/mL | 10 (47.6) | 27 (33.8) |  |
| >10^3^/mL | 8 (38.1) | 49 (61.3) |  |
| N/A | 3 (14.3) | 4 (5) |  |
| BCLC stage, n (%) |  |  | 1 |
| C | 21 (100) | 80 (100) |  |
| CNLC stage, n (%) |  |  | 0.611 |
| IIIa | 17 (81) | 58 (72.5) |  |
| IIIb | 4 (19) | 22 (27.5) |  |
| Extrahepatic disease, n (%) |  |  | 0.611 |
| No | 17 (81) | 58 (72.5) |  |
| Yes | 4 (19) | 22 (27.5) |  |
| AFP, ng/mL, median (IQR) | 2956 (114, 7585) | 1871.5 (21.9, 31783.25) | 0.917 |
| AFP, n (%) |  |  | 0.839 |
| ≤400 ng/mL | 7 (33.3) | 31 (38.8) |  |
| >400 ng/mL | 14 (66.7) | 49 (61.3) |  |
| PIVKA-II, mAU/mL, median (IQR) | 15970 (1029, 55532) | 8820 (886, 35163.5) | 0.669 |
| PIVKA-II, n (%) |  |  | 1 |
| ≤1000 mAU/mL | 5 (23.8) | 21 (26.2) |  |
| >1000 mAU/mL | 16 (76.2) | 59 (73.8) |  |
| Diameter of intrahepatic tumors, median (IQR) | 11.53 ± 5.86 | 11.64 ± 5.09 | 0.935 |
| Treatment line, n (%) |  |  | 1 |
| 1 | 20 (95.2) | 72 (90) |  |
| 2 | 1 (4.8) | 7 (8.8) |  |
| 3 | 0 (0) | 1 (1.2) |  |
| Anti-PD-1 antibody used, n (%) |  |  | 0.985 |
| Camrelizumab | 8 (38.1) | 26 (32.5) |  |
| Nivolumab | 1 (4.8) | 7 (8.8) |  |
| Pembrolizumab | 3 (14.3) | 9 (11.2) |  |
| Sintilimab | 7 (33.3) | 28 (35) |  |
| Tislelizumab | 1 (4.8) | 6 (7.5) |  |
| Toripalimab | 1 (4.8) | 4 (5) |  |
| BOR per RECIST v1.1, n (%) |  |  | 0.528 |
| CR | 0 (0) | 2 (2.5) |  |
| PR | 4 (19) | 19 (23.8) |  |
| SD | 8 (38.1) | 38 (47.5) |  |
| PD | 9 (42.9) | 21 (26.2) |  |
| Objective response per RECIST v1.1, n (%) |  |  | 0.692 |
| No | 17 (81) | 59 (73.8) |  |
| Yes | 4 (19) | 21 (26.2) |  |
| Disease control per RECIST v1.1, n (%) |  |  | 0.225 |
| No | 9 (42.9) | 21 (26.2) |  |
| Yes | 12 (57.1) | 59 (73.8) |  |
| BOR per mRECIST, n (%) |  |  | 0.527 |
| CR | 1 (4.8) | 4 (5) |  |
| PR | 5 (23.8) | 26 (32.5) |  |
| SD | 6 (28.6) | 29 (36.2) |  |
| PD | 9 (42.9) | 21 (26.2) |  |
| Objective response per mRECIST, n (%) |  |  | 0.614 |
| No | 15 (71.4) | 50 (62.5) |  |
| Yes | 6 (28.6) | 30 (37.5) |  |
| Disease control per mRECIST, n (%) |  |  | 0.225 |
| No | 9 (42.9) | 21 (26.2) |  |
| Yes | 12 (57.1) | 59 (73.8) |  |
| Change from baseline per RECIST v1.1, median (IQR) | -0.16 (-0.19, -0.02) | -0.11 (-0.33, 0) | 0.786 |
| Change from baseline per mRECIST, median (IQR) | -0.32 (-0.63, -0.09) | -0.26 (-0.53, 0) | 0.41 |
| Surgery after therapy, n (%) |  |  | 0.067 |
| No | 21 (100) | 68 (85) |  |
| Yes | 0 (0) | 12 (15) |  |

*AFP* α-fetoprotein, *BCLC* Barcelona Clinic liver cancer, *BOR* best overall response, *CNLC* China liver cancer, *CR* complete response, *ECOG PS* Eastern Cooperative Oncology Group performance status, *HBsAg* hepatitis B surface antigen, *HBV* hepatitis B virus, *IQR* interquartile range, *mRECIST* modified Response Evaluation Criteria in Solid Tumors, *N/A* not available, *PD-1* programmed death-1, *PD* progressive disease, PIVKA-II protein induced by vitamin K absence or antagonist-II, *PR* partial response, *RECIST* Response Evaluation Criteria in Solid Tumors, *SD* stable disease.

**Supplementary Table 5. Comparison of clinicopathologic features and tumor responses between patients with or without extrahepatic metastasis**

| **Variables** | **Without extrahepatic metastases**  **(n = 112)** | **With extrahepatic metastases**  **(n = 75)** | ***P* value** |
| --- | --- | --- | --- |
| Age, years, mean ± standard deviation | 56.3 ± 11.17 | 53.69 ± 11.63 | 0.129 |
| Sex, n (%) |  |  | 1 |
| Female | 14 (12.5) | 9 (12) |  |
| Male | 98 (87.5) | 66 (88) |  |
| ECOG PS, n (%) |  |  | 0.203 |
| 0–1 | 108 (96.4) | 69 (92) |  |
| 2 | 4 (3.6) | 6 (8) |  |
| Child-Pugh class, n (%) |  |  | 0.267 |
| A | 105 (93.8) | 66 (88) |  |
| B | 7 (6.2) | 9 (12) |  |
| HBsAg, n (%) |  |  | 0.201 |
| Negative | 16 (14.3) | 17 (22.7) |  |
| Positive | 96 (85.7) | 58 (77.3) |  |
| HBV DNA, n (%) |  |  | 0.464 |
| ≤10^3^/mL | 51 (45.5) | 36 (48) |  |
| >10^3^/mL | 51 (45.5) | 27 (36) |  |
| N/A | 10 (8.9) | 12 (16) |  |
| BCLC stage, n (%) |  |  | **<0.001** |
| B | 37 (33) | 0 (0) |  |
| C | 75 (67) | 75 (100) |  |
| CNLC stage, n (%) |  |  | **<0.001** |
| IIb | 37 (33) | 0 (0) |  |
| IIIa | 75 (67) | 0 (0) |  |
| IIIb | 0 (0) | 75 (100) |  |
| Macrovascular invasion, n (%) |  |  | **<0.001** |
| No | 37 (33) | 49 (65.3) |  |
| Yes | 75 (67) | 26 (34.7) |  |
| AFP, ng/mL, median (IQR) | 607 (16.48, 11472.5) | 1104 (8.85, 26506.5) | 0.791 |
| AFP, n (%) |  |  | 0.631 |
| ≤400 ng/mL | 50 (44.6) | 37 (49.3) |  |
| >400 ng/mL | 62 (55.4) | 38 (50.7) |  |
| PIVKA-II, mAU/mL, median (IQR) | 3962 (371.75, 25832) | 2984 (142.5, 21934) | 0.374 |
| PIVKA-II, n (%) |  |  | 0.526 |
| ≤1000 mAU/mL | 37 (33) | 29 (38.7) |  |
| >1000 mAU/mL | 75 (67) | 46 (61.3) |  |
| Diameter of intrahepatic tumors, median (IQR) | 9.9 (5.8, 14.45) | 8.5 (2.78, 14.35) | 0.185 |
| Treatment line, n (%) |  |  | 0.076 |
| 1 | 97 (86.6) | 56 (74.7) |  |
| 2 | 14 (12.5) | 18 (24) |  |
| 3 | 1 (0.9) | 1 (1.3) |  |
| Anti-PD-1 antibody used, n (%) |  |  | 0.149 |
| Camrelizumab | 37 (33) | 22 (29.3) |  |
| Nivolumab | 7 (6.2) | 4 (5.3) |  |
| Pembrolizumab | 16 (14.3) | 5 (6.7) |  |
| Sintilimab | 41 (36.6) | 27 (36) |  |
| Tislelizumab | 4 (3.6) | 9 (12) |  |
| Toripalimab | 7 (6.2) | 8 (10.7) |  |
| BOR per RECIST v1.1, n (%) |  |  | 0.345 |
| CR | 3 (2.7) | 1 (1.3) |  |
| PR | 22 (19.6) | 23 (30.7) |  |
| SD | 56 (50) | 31 (41.3) |  |
| PD | 31 (27.7) | 20 (26.7) |  |
| Objective response per RECIST v1.1, n (%) |  |  | 0.192 |
| No | 87 (77.7) | 51 (68) |  |
| Yes | 25 (22.3) | 24 (32) |  |
| Disease control per RECIST v1.1, n (%) |  |  | 1 |
| No | 31 (27.7) | 20 (26.7) |  |
| Yes | 81 (72.3) | 55 (73.3) |  |
| BOR per mRECIST, n (%) |  |  | 0.422 |
| CR | 7 (6.2) | 2 (2.7) |  |
| PR | 32 (28.6) | 29 (38.7) |  |
| SD | 42 (37.5) | 24 (32) |  |
| PD | 31 (27.7) | 20 (26.7) |  |
| Objective response per mRECIST, n (%) |  |  | 0.455 |
| No | 73 (65.2) | 44 (58.7) |  |
| Yes | 39 (34.8) | 31 (41.3) |  |
| Disease control per mRECIST, n (%) |  |  | 1 |
| No | 31 (27.7) | 20 (26.7) |  |
| Yes | 81 (72.3) | 55 (73.3) |  |
| Change from baseline per RECIST v1.1, median (IQR) | -0.13 (-0.27, 0) | -0.18 (-0.39, 0) | 0.322 |
| Change from baseline per mRECIST, median (IQR) | -0.24 (-0.56, 0) | -0.35 (-0.68, 0) | 0.598 |
| Surgery after therapy, n (%) |  |  | 0.641 |
| No | 93 (83) | 65 (86.7) |  |
| Yes | 19 (17) | 10 (13.3) |  |

*AFP* α-fetoprotein, *BCLC* Barcelona Clinic liver cancer, *BOR* best overall response, *CNLC* China liver cancer, *CR* complete response, *ECOG PS* Eastern Cooperative Oncology Group performance status, *HBsAg* hepatitis B surface antigen, *HBV* hepatitis B virus, *IQR* interquartile range, *mRECIST* modified Response Evaluation Criteria in Solid Tumors, *N/A* not available, *PD-1* programmed death-1, *PD* progressive disease, PIVKA-II protein induced by vitamin K absence or antagonist-II, *PR* partial response, *RECIST* Response Evaluation Criteria in Solid Tumors, *SD* stable disease.
